# Nitrogen and phosphorus stoichiometry of *Schima superba* under nitrogen deposition

**DOI:** 10.1038/s41598-018-32031-y

**Published:** 2018-09-12

**Authors:** Rui Zhang, Hongwei Pan, Biting He, Huanwei Chen, Zhichun Zhou

**Affiliations:** 1 Research Institute of Subtropical Forestry, CAF. Zhejiang Provincial Key Laboratory of Tree Breeding. Daqiao road73#, Fuyang, 311400 Zhejiang, PR China; 2 Longquan Academy of Forestry, Zhejiang, 323700 China

## Abstract

In this study, leaf nitrogen (N) and phosphorus (P) stoichiometry were used as indicators of nitrogen saturation and to assess ecosystem nutrient limitations. *Schima superba*, a representative and widely distributed dominant evergreen broadleaf tree species of the subtropical forests in southern China, was used for this purpose. A nutrient-addition experiment and a field survey were conducted to test the responses of trees from different provenances to N deposition. The relationships between leaf N and P stoichiometry and biomass, nutrient limitation, and soil N:P were analyzed. There was a relationship between leaf N, P, N:P, soil N:P and plant dry biomass. A threshold leaf N:P ratio (16.3) divided the five provenances into different nutrient-limitation classes that were related to the soil N:P ratio or N deposition. The leaf N:P ratio provided an indication of P limitation. A higher soil P level reduced the N deposition effect on plant growth. The leaf N:P ratio of individuals from different provenances can be used as a predictor of nutrient limitation, and this was related to the soil N:P ratio.

## Introduction

The nitrogen (N) and phosphorous (P) levels in plant tissues, especially in the leaves, have often been studied, and the N:P ratio is considered an important indicator of nutrient limitation within individual species as well as for the entire ecosystem^1–8^. In wetland ecosystems, the leaf N:P ratio is <14 under N-limited conditions and >16 under P-limited conditions^9^. However, in upland ecosystems, the threshold value of the leaf N:P ratio varies considerably (ranging from 6.7 to 16 under N-limited conditions and 12.5 to 26.3 under P-limited conditions), and vegetation growth is limited by P^10^. Numerous studies have shown that leaf N and P stoichiometry is affected by factors such as habitat, functional group, and growth stage^4–6,11–13^. An important environmental factor influencing plant biomass and the leaf N:P ratio is the N:P ratio of the soil solution^4,7^. A species that occurs in different areas could have different leaf N:P ratios based on soil N:P ratio variation^14^. In southern China, a higher leaf N:P ratio occurs in woody plants in response to a shortage of soil P^6,13^.

**Figure 1:**
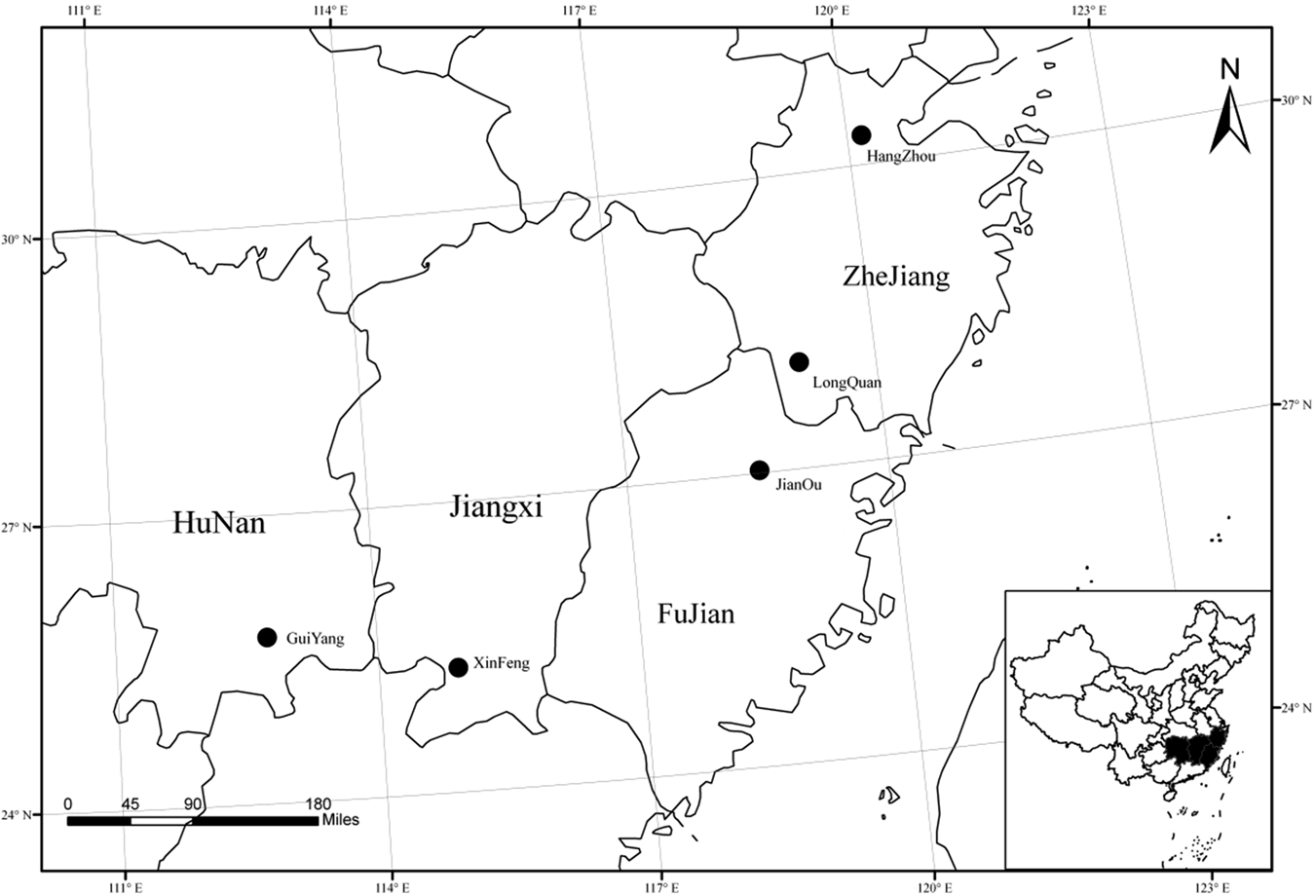
Geographical distribution of the study provenances of *S*. *superba* in southern China.

N deposition is high in the subtropical forests of southern China. Zhu *et al*. reported that the average deposition fluxes of N and P in China were 13.69 ± 8.69 kg ha^−1^ a^−1^ and 0.21 ± 0.17 kg ha^−1^ a^−1^, respectively^8^. The N:P ratios of atmospheric wet deposition (average = 77 ± 40) were found to be negatively correlated with current soil N:P ratios. In addition to the low soil P concentration, such N:P ratio imbalances will disturb nutrient availability and increase P limitation^8,15^. This deposition trend is expected to increase in future decades, and the production of reactive N will reach 270 Tg N a^−1^ by 2050^8,16–22^. We evaluated how a widespread species of different provenances would respond to soil N:P ratios and determined the biomass patterns corresponding to the N:P ratios of this species under N deposition in China. *Schima superba*, a representative and widely distributed broadleaf evergreen tree species of subtropical forests in southern China, was used as the study material^23^. Based on uniform levels of simulated N deposition in several provenances of this species, we found that the root system was more developed, and P absorption efficiency (PAE) was higher under N deposition, which was related to soil nutrition^24^. From these results, we inferred that there would be a relationship between biomass and the leaf N:P ratios and that changes would occur in the leaf N and P stoichiometry patterns in this species under N deposition. We also inferred that leaf N:P in *S*. *superba* could be a predictor of nutrient limitation in nursery breeding or in forest management. Our objectives were to (a) compare the seedling growth, leaf N, P, and N:P ratio in *S*. *superba* from different provenances in P-limited and P-rich soil under simulated N deposition treatments; (b) determine whether seedlings from different provenances with different N and P availability exhibited the same nutrient limitation; (c) compare the N:P ratio of different forest sites (Table 1, Fig. 1) using results of (a); and (d) determine the relationship between the leaf N:P ratio, the soil N:P ratios, and the biomass of *S*. *superba*, and assess N deposition effects on leaf N and P stoichiometry.

**Table 1:** Ecological and geographical parameters of five *S*. *superba* forest sites and the average biomass per plant [means with standard errors (S.E.) in parentheses, n = 30 for all samples]*.

Forest sites	Distribution areas	Longitude (E)/Latitude (N)	Mean annual temperature (°C)	Mean annual precipitation (mm)	N deposition (2007) (kg ha^−1^ year^−1^)	Forest age (year)	Average DBH (cm)	Average Height (m)	Average Biomass (kg)	Biomass per year (kg/year)
HZ-F	Zhejiang	119.1 (E)/29.5 (N)	16.2	1435	>45	12	15.13 (1.11)c	11.5 (0.74)b	70.8 (13.6)c	5.9 (1.1) b
LQ-F	Zhejiang	119.1 (E)/27.4 (N)	15.3	1900	32.4	30	19.19 (2.13)b	18.4 (2.38)a	184.2 (57.1)a	6.1 (1.9) b
JO-F	Fujian	118.3 (E)/27.1 (N)	18.7	1723	27.8	17	21.57 (3.69)a	10.9 (1.22)b	137.4 (65.2)b	8.1 (2.8) a
XF-F	Jiangxi	114.7 (E)/27.8 (N)	19.2	1510	22.2	23	18.99 (3.36)b	14.7 (1.49)a	143.7 (64.4)b	6.3 (2.6) b
GY-F	Hunan	112.7 (E)/25.8 (N)	17.3	1437	17.5	28	21.64 (3.21)a	13.6 (1.87)ab	173.0 (74.2)ab	6.2 (2.2) b

*The ecological and geographical parameters were obtained from meteorological stations, and the forest data were obtained from the forestry station. Values followed by different letters within a column indicate significant differences between sites. p < 0.05. The average biomass is the dry weight of the aboveground components, and the formula used was $W=0.0245{({D}^{2}H)}^{1.0118}$. D represents the diameter at breast height and H represents plant height^51^.

## Results

### Leaf N and P concentrations and the N:P ratios under N deposition

There was no interaction between provenances and nutrient input level, and only main effect responses for all variables were observed under the experimental treatment (Table 2). Regardless of the P effect, the leaf N concentration (16.8 mg g^−1^, *p* < 0.05) and N:P ratio (19.5, *p* < 0.05) in the N200 treatment were significantly higher than in the other three treatments (N0: 10.0 mg g^−1^ and 9.9; N50: 10.2 mg g^−1^ and 13.1; N100: 12.4 mg g^−1^ and 17.0, respectively). However, the highest leaf P concentration was measured in the N0 treatment (1.06 mg g^−1^) compared to N50 (0.86 mg g^−1^); N100 (0.88 mg g^−1^), and N200 (0.77 mg g^−1^). The biomass, leaf P concentration, and N:P ratios all significantly differed among provenances, P, and N treatments. The leaf N did not differ significantly among the provenances (Tables 2, 3).

**Table 2:** ANOVA for comparisons of aboveground biomass (AB), leaf N (LN), P (LP), and the leaf N:P ratio (L N:P) of *S*. *superba* between treatments in the N deposition experiment, i.e., four N levels and two P levels (n = 192).

	F value			
	AB	LN	LP	L N:P
N	21.11**	37.34**	17.74**	51.81**
P	82.99**	106.83**	302.43**	7.54**
Provenance	8.49**	0.60	4.77**	3.45**
N × P	5.40**	14.57**	2.38	3.13*
N × Provenance	1.37	0.59	0.50	0.50
P × Provenance	0.44	0.53	0.42	1.03
N × P × Provenance	2.16	0.52	0.29	0.71

The degrees of freedom for N, P, provenance, N × P, N × provenance, P × provenance, and N × P × provenance were 3, 1, 4, 3, 12, 2, and 6, respectively. **p ≤ 0.01, *0.01 < *p* < 0.05.

**Table 3:** Aboveground biomass (AB), leaf nitrogen (LN) and phosphorus (LP) concentrations, and N:P ratios (L N:P), and the soil nitrogen (SN) and phosphorus (SP) concentrations and N:P ratios (S N:P) of the five provenances of *S*. *superba* in the N deposition experiment (means with S.E. in parentheses, n = 192 for plant samples and n = 8 for soil samples).

Provenance	AB (g)	LN (mg g^−1^)	LP (mg g^−1^)	L N:P	SN (g kg^−1^)	SP (g kg^−1^)	S N:P ratio
HZ	4.5 (1.2)ab	13.67 (7.4)a	1.04 (0.3)a	13.6 (6.1)b	0.40 (0.03)ns	0.23 (0.07)a	1.84 (0.48)ab
LQ	5.3 (1.1)ab	12.96 (5.4)ab	1.00 (0.3)a	14.0 (5.2)b	0.41 (0.02)ns	0.25 (0.03)a	1.79 (0.58)b
JO	5.9 (1.1)a	13.07 (6.5)ab	0.92 (0.4)a	14.9 (5.1)ab	0.43 (0.05)ns	0.26 (0.08)a	1.78 (0.51)b
XF	4.1 (0.7)bc	9.01 (1.9)c	0.59 (0.2)b	16.5 (4.7)ab	0.41 (0.02)ns	0.18 (0.07)b	2.30 (0.55)a
GY	3.3 (0.9)c	10.36 (3.1)bc	0.63 (0.2)b	17.4 (5.3)a	0.41 (0.02)ns	0.18 (0.03)b	2.30 (0.58)a

Values followed by different letters within a column indicate significant differences between sites. ns: no significant difference.

The leaf N, P and N:P ratios exhibited large variations across forest sites, ranging from 13–18 mg g^−1^ for N, 0.2–0.4 mg g^−1^ for P, and 35–85 for the N:P ratios (Table 4). The mean values of the leaf N, P and N:P ratio for all forest sites were 14.7 mg g^−1^, 0.34 mg g^−1^, and 50.0, respectively.

**Table 4:** Leaf nitrogen (LN) and phosphorus (LP) concentrations and N:P ratio (L N:P), and the soil nitrogen (SN) and phosphorus (SP) concentrations and soil N:P ratios (S N:P) measured in the five *S*. *superba* forest sites (means with S.E. in parentheses, n = 30 for plant samples and n = 5 for soil samples)

Forest site	Distribution area	LN (mg g^−1^)	LP (mg g^−1^)	L N:P	SN (g kg^−1^)	SP (g kg^−1^)	S N:P
HZ-F	Zhejiang	18.03 (3.76)a	0.24 (0.11)b	85.3 (27.1)a	1.79 (0.77)a	0.62 (0.33)b	2.92 (1.08)a
LQ-F	Zhejiang	13.71 (2.06)b	0.32 (0.07)ab	44.9 (11.2)b	0.92 (0.36)c	0.36 (0.17)c	2.63 (0.83)b
JO-F	Fujian	15.56 (2.72)ab	0.35 (0.07)a	46.5 (10.7)b	1.34 (0.55)b	0.93 (0.46)a	1.52 (0.80)c
XF-F	Jiangxi	13.16 (3.38)b	0.39 (0.12)a	37.5 (14.5)b	1.25 (0.33)bc	0.68 (0.11)b	1.90 (0.76)bc
GY-F	Hunan	13.19 (2.63)b	0.42 (0.12)a	35.5 (15.7)b	1.17 (0.59)bc	0.54 (0.36)c	2.22 (0.91)bc

Values followed by different letters within a column indicate significant differences between sites.

The leaf N:P ratios increased with N deposition level both in the experimental treatments (Table 3) and forest field sites (Table 4).

### Relationship between biomass and leaf N, leaf P, and N:P ratio

Both aboveground biomass and total biomass were significantly positively correlated with leaf N and P, and they were more strongly correlated with leaf N under experimental N deposition treatment (Table 5). The aboveground biomass and the leaf N:P ratio had a correlation coefficient of 0.44, *p* < 0.001 (Table 5), and a regression curve (W_aboveground Biomass_ = −0.014(ratio_N:P_)^2^ + 0.4552 ratio_N:P + _0.4318, R^2^ = 0.2958, *p* < 0.001, n = 192) was fitted. The highest biomass value was measured at a leaf N:P ratio of 16.3.

**Table 5:** Correlation coefficients between aboveground biomass (AB), total biomass (TB), leaf nitrogen (LN), phosphorus (LP), leaf N:P ratio (L N:P) (n = 192), and soil N:P (S N:P) in the N deposition experiment.

	r		r
LN-LP	0.43**		
AB-LN	0.75**	TB-LN	0.69**
AB-LP	0.30**	TB-LP	0.44**
AB-LN:P	0.44**	TB-LN:P	0.28**
AB-SN:P (n = 60)	−0.60**	TB-SN:P (n = 60)	−0.79**

The correlation coefficient (r) between leaf N and P was calculated for each treatment. **p < 0.001.

Although the field forest leaf N:P ratio was higher than that recorded in the experimental N deposition study, there was a similar trend between the biomass and the leaf N:P ratio (Tables 1, 4).

The aboveground biomass was significantly negatively correlated with the soil N:P ratio and the correlation coefficient was −0.6, *p* < 0.001 (Table 5). The relationship between the forest field biomass and the soil N:P (Table 3) was the same as that shown for the experimental results (Table 4). The five provenances in the experiment had a higher aboveground biomass when the soil N:P value was less than 2. JO had the highest biomass, which was associated with a leaf N:P ratio of 14.9 (Table 3). In the forest field site, JO-F also had the highest biomass, and its soil N:P ratio was only 1.5 (Table 4).

The principal components model using nutrient element, biomass, and environmental variables from five forest sites established four main principal components with a degree of explanation >87% (Table 6). The first component explained 41.10% of the variability, the second component explained 21.42%, the third one explained 14.61%, and the fourth one explained 10.12% (Table 6). Principal component 1 was related to leaf N:P, sample sites, N deposition, and biomass (Table 7). Soil N:P was correlated with principal component 2, and it was correlated statistically (*p* < 0.05) with annual temperature and tree height (Table 7).

**Table 6:** Statistical parameters of the principal components from the N, P, N:P ratios, biomass, and environmental variables in *S*. *superba* forest sites (n = 30 for plant samples and n = 5 for soil samples).

Parameters	Principal component			
	1	2	3	4
Eigenvalue	6.987	3.64	2.48	1.72
% Total of variance	41.10	21.42	14.61	10.21
Total cumulative	41.10	62.52	77.13	87.25

**Table 7:** Correlations between different principal components and N, P, N:P ratios, biomass, and environmental variables in *S*. *superba* forest sites (n = 30 for plant samples and n = 5 for soil samples)

Parameters	Principal component			
	1	2	3	4
N deposition	0.91**	0.29	0.12	0.06
longitude	0.74**	0.31	−0.12	0.53**
latitude	0.90**	0.00	0.07	−0.10
Mean annual precipitation	−0.07	0.47	−0.47	0.73**
Mean annual temperature	−0.30	−0.90**	−0.02	0.12
Leaf N/P	0.76**	0.14	0.42	0.01
Leaf N	0.44	0.01	0.61**	0.22
Leaf P	−0.61**	−0.24	−0.21	−0.17
Soil N/P	0.48	0.68**	0.17	−0.51**
Soil N	0.72**	−0.43	0.45	−0.24
Soil P	0.12	−0.88**	0.13	0.42
Age	−0.76**	0.52**	−0.33	−0.07
DBH	−0.79**	0.02	0.52**	0.26
Height	−0.48	0.73**	0.31	0.08
Average biomass	−0.74**	0.31	0.55**	0.11
Biomass per year	−0.53**	0.09	0.77**	0.25
Crown width	−0.73**	−0.19	0.17	−0.39

*p < 0.05; **p < 0.01, statistically significant.

## Discussion

### Leaf N, P, and N:P under N deposition

We conducted an analysis of the leaf N and P stoichiometry from five different provenances inhabited by *S*. *superba*, a widely distributed and dominant subtropical broad-leaved evergreen in southern China.

Leaf N and leaf N:P ratio were increased but leaf P was decreased with the addition of N. This result is similar to the results of Mo *et al*. for two species (*S*. *superba* and *Cryptocarya concinna*) in China^18^. Increases in N availability under N deposition are typically associated with increases in plant N concentration^25,26^ and decreases in P concentration^27–30^. This is related to the increase in N supply, and the greater N uptake results in an increased uptake of other nutrients such as P. However, these other nutrients may become limiting over time. After long-term N deposition, the concentration of P, Ca, Mg, or other cations in soils tends to decrease^28,30^, and deficiency of these elements in plants is often observed^14,29–33^. Chronic increases in N deposition increase the soil and leaf N:P ratios, limiting plant growth^24,34–36^ and photosynthetic rates^32,37^ and leading to less diverse ecosystems. The abundance of nitrophilous species may increase^38^. A higher soil P concentration could reduce the negative effect of N deposition, increase the leaf N and P concentration, and decrease the leaf N:P (Low-P: W_AB_ = 2.5 g, LN = 9.8 mg g^−1^, LP = 0.7 mg g^−1^, L N:P = 15.5; High-P: W_AB_ = 4.5 g, LN = 16.6 mg g^−1^, LP = 1.3 mg g^−1^, L N:P = 13.8). The leaf N:P increased from the southwest to the northeast. The soil P and N:P were related to temperature in principal component 2 (Table 7). The soil P concentration increases from the southeastern coastal areas to the inland areas of southern China^39,40^, and this is due to temperature leading to soil P decomposition by weathering. Therefore, soil P concentration is an important factor affecting plant development, especially plant height, and it reflects a bio-geographical gradient pattern at local scales^2,13^.

A previous study found that leaf N and P stoichiometry could be used as indicators of nutrient limitation in the ecosystem^1–8^. We found that the leaf N:P ratio was a good predictor of nutrient limitation of individual species such as *S*. *superba*. Young seedlings in the experiment had a leaf N:P ratio (15.3) that was similar to 149 evergreen tree species (15.2) and 255 broadleaf tree species (15.1) in China^13^, and 74 evergreen woody species (13.4) worldwide^41^. This suggested that the leaf N:P was weakly affected by the seedling age and species difference and had a similar value as large categories of woody species^42^. However, in our forest site survey, the average value of the leaf N:P ratio was 49.9, which is 3.4 times greater than the average value found in previous studies. A possible cause of the substantially higher leaf N:P ratio may be the different soil and growth conditions of *S*. *superba*. Roughgarden predicted that the level of phenotypic variance is often associated with niche width^43^. Forest ecosystems have higher N and P deposition ratios (91:1) than other ecosystem types^8^. At the same time, larger crowns and greater leaf areas intercept more N from N deposition, and *S*. *superba* growing in forest sites absorbs more inorganic N through the leaves^44^. We found that the soil N concentration was high in the five forest sites, and this may be maintained by a deep litter layer and high organic matter content (Table 3). Thus, high levels of inorganic N and leaching from foliar tissue or directly from N deposition may be transported into the soil and lead to soil N saturation. In addition, an increased N supply and uptake can result in the soil deficiency of P and other elements^14,28–32^. This means that niche size, with respect to nutrient cycling, would be in the range of mineral element concentrations in environments in which the species can grow and reproduce^42^. The correlation and principal component analysis between leaf N:P and the environment confirmed that N deposition had a considerable effect on leaf N:P (r^2^ = 0.874, *p* = 0.019), and the leaf N:P ratio can be used as an indicator for individual species to assess the degree of N deposition (Table 7).

### Leaf N:P, biomass, and nutrient limitation threshold

The threshold value indicated by the regression curve between the biomass and leaf N:P ratio was 16.3. When the leaf N:P ratio was lower than this value, plant growth was limited by N. In contrast, at a N:P ratio higher than 16.3, plant growth was limited by P. Leaf N:P ratios were considerably higher in the forest field sites, and these trees were limited by P. This may be caused by the very low available soil P in southern China^36,37^. However, different results were obtained from the experiment using one-year seedlings originating from the same provenances. For example, N limitations were becoming evident in seedlings from HZ, LQ, and JO (the leaf N:P ratio was lower than 16.3) (Table 2). N is present in forms such as amino acids or proteins and thus incurs a metabolic cost for storage^45^. Therefore, plants with rapid N metabolism undergo constant turnover, which creates a large N demand for plant growth^46^. N deposition satisfies the N demand, and trees growing in areas under long-term and high N deposition were found to have faster turnover^47^. If these plants had grown in areas without N deposition, they may have been N limited.

The extent to which the plant N:P ratio reflects that of the soil solution is a function of the homeostatic regulation exerted by the plant over its N:P stoichiometry^4,7^. For example, there was a greater increase in plant biomass in response to P addition compared with N addition. This suggested that the growth of *S*. *superba* at the five forest sites was limited by P-deficiency. Aerts and Chapin showed that P-deficient plants increase their P uptake rate and reduce the rate of N uptake to regulate the leaf N:P ratio, whereas N-deficient plants reduce the P uptake rate and increase the N uptake rate to maintain stoichiometric balance^2^. Therefore, there is a complex relationship between the soil N:P and the leaf N:P, and both of these ratios affect plant biomass. Based on the relationship between soil N:P and biomass, we found that when the soil N:P ratio was less than 1.8, the plant biomass increased. However, when this ratio was higher than 2.3, biomass was lower (Tables 1, 3 and 4). This suggests that a range of soil N:P can improve plant growth, and 1.8 may be an important threshold value for *S*. *superba*. Zhu *et al*. provided a formula for soil N:P ratios and atmospheric deposition N:P ratios, which was: N:P_soil_ = −0.0108 N:P_atmospher_ + 3.3552 (R^2^ = 0.8, P = 0.007)^8^. We also propose that atmospheric deposition N:P ratios ranging from 97.7 to 144 would promote *S*. *superba* growth. Further studies are required to test this hypothesis.

## Conclusions

We found that a higher soil P concentration could weaken the negative effect of N deposition. Plant leaf N:P ratios of *S*. *superba* from different provenances were affected by habitat, and this ratio could be an effective predictor of the nutrient limitation level of the habitat. Leaf N:P could be used as an nutrient index in tree breeding in addition to its traditional ecological significance. Soil N:P ratios had a complex relationship with leaf N:P and they were found to affect plant biomass. These results can guide the selection of optimal nutrient values for nursery tree breeding and forest management.

## Materials and Methods

This study was conducted by the Forestry Genetic and Breeding Lab at the Research Institute of Subtropical Forestry, CAF, State Forestry Administration, China (RISF-CAF). The State Forestry Administration is responsible for national parks and other protected areas. No specific permission was required for these locations/activities, as they were based on non-destructive collection of plant material. The species is not endangered or protected, and the locations were not privately owned or protected by law.

### Determination of the plant N:P ratio in forests

The five study sites for this project were in natural forests or plantations of *S*. *superba*. They were Hangzhou (HZ-F) and Longquan in Zhejiang province (LQ-F), Jianou in Fujian province (JO-F), Xinfeng in Jiangxi province (XF-F), and Guiyang in Hunan province (GY-F). The forest ages ranged from 12 to 30 years (Table 1). The N deposition in these areas over an approximate 30-year period (from 1980 to 2007) was 10–20 kg ha^−1^ a^−1^ in Hunan, 20–30 kg ha^−1^ a^−1^ in Jiangxi, 30–45 kg ha^−1^ a^−1^ in Fujian, and more than 45 kg ha^−1^ a^−1^ in Zhejiang province^20^ (Table 1, Fig. 1). The mature leaf samples for N and P analysis in this study were randomly collected from 30 trees in each of the forest sites in June 2017. The samples collected were the third or fourth leaf from the top of the current year branch, and 10 leaves were collected per tree. Soil samples (1 kg, collected from a 0–20 cm depth), collected from five different locations in each forest site, were pooled. The plant material and soil were dried and sieved through a 1 mm sieve to determine the mineral composition of the samples. They were then digested with H_2_SO_4_ and the catalyst H_2_O_2_, and diluted with deionized water to 50 mL after cooling. Total N was determined using the standard Kjeldahl method^48^. Total P was determined using the Mo-Sb anti-spectrophotography method^49^.

### Experimental assessment

The experiment included five provenances (HZ, LQ, JO, XF, and GY) of *S*. *superba* seedlings, P-limited soil, and four levels of N addition using NH_4_NO_3_. The levels were N0 = 0 kg N ha^−1^ year^−1^, N50 = 50 kg N ha^−1^ year^−1^, N100 = 100 kg N ha^−1^ year^−1^, and N200 = 200 kg N ha^−1^ year^−1^. These N levels were determined based on current and historical N deposition in southern China^8,17,19,20,50^. The comparison set consisted of three provenances (HZ, JO, and XF), P-rich soil, and four levels of N addition. There were 12 replicates per treatment, with a total of 384 individuals. In November 2017, six plants were randomly chosen from each treatment; these plants were harvested and their biomass was determined. The soil in the six containers was mixed and the composition was determined.

The P-limited soil (P-L) used in this experiment was regular forest soil obtained from Zhejiang province. The pH value of this soil was 5.06 (potentiometric method, LY/T 1239–1999); total N was 0.41 g kg^−1^ (semi-micro Kjeldahl method, LY/T 1228–1999), hydrolyzable N was 46.43 mg kg^−1^ (alkaline hydrolysis diffusion method, LY/T 1229–1999), available P was 13 mg kg^−1^ (hydrochloric acid-sulfuric acid extraction method, LY/T 1233–1999), available potassium (K) was 130.08 mg kg^−1^ (1 mol L^−1^ ammonium acetate extraction–flame photometry method, LY/T 1236–1999), and organic matter was 6.68 g kg^−1^ (potassium dichromate oxidation–external heating method, LY/T 1237–1999). The P-rich soil (P-H) received KH_2_PO_4_ until the available P measured 60 mg kg^−1^.

The N deposition experiment was carried out from March to November 2017 in a glass house without climate control. The temperature in the glass house ranged from 5 °C to 15 °C from December to the following February and 26 °C to 34 °C during the period from July to September 2017. The *S*. *superba* seeds from the five provenances were germinated in March 2017. The seeds were collected from Hangzhou (HZ) and Longquan (LQ) in Zhejiang province, Jianou (JO) in Fujian province, Guiyang (GY) in Hunan province, and Xinfeng (XF) in Jiangxi province (Table 1). When the seedlings had two cotyledons and were 2–3 cm tall, they were planted in plastic containers (15 cm diam. × 18 cm high) in April 2017, with one seedling per container. A NH_4_NO_3_ solution was sprayed from the foliage to the soil once every two weeks for one month, and once a month for the next six months. The spraying began on 20^th^ April 2017 when the leaves started to bud. The concentrations of the NH_4_NO_3_ solution were 0.0016 mol L^−1^ for N50, 0.0031 mol L^−1^ for N100 and 0.0062 mol L^−1^ for N200. The seedlings were watered every two days with distilled water until they were harvested during the first week of November 2017. Pests and weeds were controlled manually.

### Data analysis

The data were analyzed using the SAS statistical program (version V8, SAS Institute, Cary, NC) and SPSS (PASW statistics 18). The N:P ratio of each plant and the soil was determined by dividing the N concentration by the P concentration. The biomass, leaf N concentration, leaf P concentration, and N:P ratio of the different N and P treatments were analyzed for statistical differences. Three-way analysis of variance (ANOVA) using a generalized linear model (GLM) was performed at a significance level of 0.05, with P, N, and provenance as the independent factors. All variables were normally distributed and did not need to be transformed. Duncan’s multiple range test was carried out to determine whether significant (*p* < 0.05) differences existed between different levels of the N and P treatments. The correlation coefficients between biomass, leaf N and P, and the N:P ratio for each treatment were calculated (*p* < 0.001). We computed the arithmetic mean of the N:P ratio and biomass of different provenances and then fitted regression equations to determine the relationship between the N:P ratios and biomass.
